# Triple modality image reconstruction of PET data using SPECT, PET, CT information increases lesion uptake in images of patients treated with radioembolization with $^{90}Y$ micro-spheres

**DOI:** 10.1186/s40658-023-00549-4

**Published:** 2023-05-03

**Authors:** Daniel Deidda, Ana M. Denis-Bacelar, Andrew J. Fenwick, Kelley M. Ferreira, Warda Heetun, Brian F. Hutton, Daniel R. McGowan, Andrew P. Robinson, James Scuffham, Kris Thielemans, Robert Twyman

**Affiliations:** 1 National Physical Laboratory, Teddington, UK; 2 Nuclear Medicine Institute, University College London, London, UK; 3 Oxford University Hospitals NHS Foundation Trust, Oxford, UK; 4 University of Oxford, Oxford, UK; 5 Royal Surrey NHS Foundation Trust, Guildford, UK; 6 Centre for Medical Image Computing, University College London, London, UK

**Keywords:** Triple modality, PET-SPECT-CT, Iterative reconstruction, Kernel method

## Abstract

**Purpose:**

Nuclear medicine imaging modalities like computed tomography (CT), single photon emission CT (SPECT) and positron emission tomography (PET) are employed in the field of theranostics to estimate and plan the dose delivered to tumors and the surrounding tissues and to monitor the effect of the therapy. However, therapeutic radionuclides often provide poor images, which translate to inaccurate treatment planning and inadequate monitoring images. Multimodality information can be exploited in the reconstruction to enhance image quality. Triple modality PET/SPECT/CT scanners are particularly useful in this context due to the easier registration process between images. In this study, we propose to include PET, SPECT and CT information in the reconstruction of PET data. The method is applied to Yttrium-90 ($^{90}$Y) data.

**Methods:**

Data from a NEMA phantom filled with $^{90}$Y were used for validation. PET, SPECT and CT data from 10 patients treated with Selective Internal Radiation Therapy (SIRT) were used. Different combinations of prior images using the Hybrid kernelized expectation maximization were investigated in terms of VOI activity and noise suppression.

**Results:**

Our results show that triple modality PET reconstruction provides significantly higher uptake when compared to the method used as standard in the hospital and OSEM. In particular, using CT-guided SPECT images, as guiding information in the PET reconstruction significantly increases uptake quantification on tumoral lesions.

**Conclusion:**

This work proposes the first triple modality reconstruction method and demonstrates up to 69% lesion uptake increase over standard methods with SIRT $^{90}$Y patient data. Promising results are expected for other radionuclide combination used in theranostic applications using PET and SPECT.

## Introduction

Nuclear medicine imaging modalities like computed tomography (CT), single photon emission CT (SPECT) and positron emission tomography (PET) are routinely used clinically to diagnose many types of cancer. They can be used to estimate and plan the dose delivered to the tumor and to the surrounding tissues and to monitor the effect of the therapy over time. Usually, in the context of theranostics, the PET images are used for dose planning and the SPECT images are used to monitor the treatment efficacy of molecular radionuclide therapy (MRT) [1]. The images acquired with PET and SPECT are affected by resolution degradation which causes partial volume effects (PVE), and therefore, also the underestimation of activity in tumoral lesions.

The information acquired from PET and SPECT and the relevant CT image can be exploited to improve the detectability of a treated lesion or to make a more accurate estimate of after treatment dose which can make personalized dosimetry easier. A triple modality scanner can allow the acquisition of all the three modalities in parallel or sequentially. This reduces the error in the registration operation that would be required in a case of acquisition in two different scanners.

A number of different tracers or pairs of diagnostic/therapeutic tracers are used in theranostic studies for specific tumors. For example, $^{123}$I and $^{131}$I are used as diagnostic and treatment agents, respectively, for thyroid cancer, $^{68}$Ga-PSMA and $^{177}$Lu-PSMA for prostate cancer and $^{99m}$Tc-MAA and $^{90}$Y for hepatocellular carcinoma (HCC) and liver metastases [2]. In this study, the focus is on $^{90}$Y, as it potentially allows the acquisition in parallel of both PET and SPECT. In fact, $^{90}$Y emits gamma rays detectable with SPECT as well as gamma rays with enough energy to produce positron-electron pairs, which are ideal for PET acquisition. For this reason, it is the ideal candidate to investigate triple modality reconstruction.

$^{90}$Y is used clinically to treat metastatic colorectal cancer and hepatocellular carcinoma with selective internal radiation therapy (SIRT), also known as radioembolization, where a solution with $^{90}$Y micro-spheres is injected directly through the hepatic arteries [3–5].

Post-treatment imaging with SPECT, for the assessment of the radionuclide activity distribution, is made possible by the interaction of the emitted $\beta ^-$ particles and the tissues. This interaction produces bremsstrahlung photons [6–10]. bremsstrahlung radiation, however, makes scatter, attenuation and detector response modeling very challenging due to the wide energy spectrum, 50–2280 keV [11].

Different studies have investigated the use of PET as a substitute of SPECT for post-SIRT imaging and provided promising results, showing improved performance when using time of flight (ToF). Nevertheless, quantification remains challenging due to the low statistics of the PET data. In addition, activity concentration in small structures ($\leqslant$ 37 mm diameter) can be underestimated due to PVEs [12–15].

To investigate the potential quantitative and qualitative improvement of triple modality reconstruction, phantom and patient PET/SPECT/CT data using $^{90}$Y were acquired. Since PET is known to have superior resolution compared to SPECT, we believe it will provide the best result. Therefore, this study focuses on the improvement of the PET image when using PET/SPECT/CT information. Furthermore, using lower resolution information, like SPECT, to enhance a higher resolution image like PET represents an interesting problem that has never been studied. The rationale behind this is that for $^{90}$Y the PET image has extremely low statistics and an enhanced SPECT image could be of comparable quality. The SPECT image is used as a local denoising prior within similar PET-SPECT regions as well as for resolution improvement via the use of CT. Resolution improvement is also achieved via the extraction of the PET image-update features as done in previous work [16]. In previous work on dual modality imaging, different methods were proposed to exploit multi-modality information [17]. These techniques had the issue of suppressing small lesions that are unique to PET. Among these methods, the kernelized expectation maximization (KEM) [18, 19] uses a machine learning stratagem called the Kernel method. In our previous work, we have introduced an extension of KEM, the hybrid KEM (HKEM), to avoid the suppression of PET unique features via the use of the PET iterative update information [16, 20, 21]. An extension of HKEM that allows the use of multiple prior images, the multiplexing HKEM (MHKEM), was also proposed and investigated [22]. The mathematical formulation of HKEM and MHKEM, as well as the details on the hyper-parameters, can be found in [16, 22]. The HKEM algorithm has been proven useful already in different applications of nuclear medicine imaging, such as cardiovascular imaging [23–26], and cancer studies.

Recently, Marquis et al. [27] used the HKEM algorithm to reconstruct SPECT images with PET images as prior information. The study was carried out with a clinical example using $^{64}$Cu/$^{67}$Cu. In our previous work [7], the HKEM algorithm was used with CT information to improve the SPECT images of the bremsstrahlung data when PET data are not available. This method was also used for the reconstruction of the SPECT images in this study.

To reconstruct the data with triple modality information MHKEM, HKEM and KEM were used.

This work aims to demonstrate the benefits of SPECT/PET/CT information in PET image reconstruction. This concept is applied to clinical SIRT patient data. Preliminary results of the NEMA phantom were presented at the 2021 IEEE Nuclear Science Symposium and Medical Imaging Conference [28]. This manuscript is organized as follows: Sect. Methods describes the details of the data acquisition, the reconstruction settings and the data analysis. Section Results presents the results which are then discussed in Sect. Discussion. Finally, conclusions are drawn in Sect. Conclusion.

## Methods

### Phantom data

**Fig. 1:**
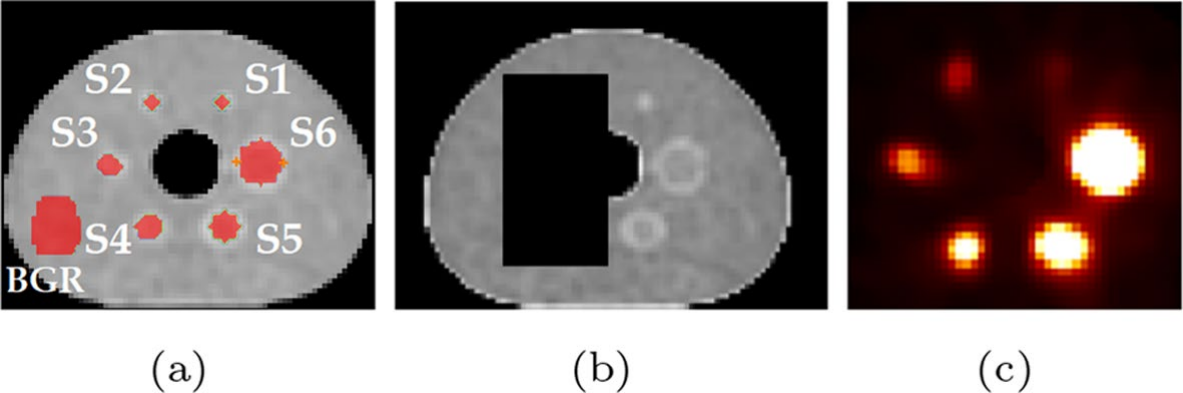
CT image with the chosen VOIs for the phantom NEMA (**a**), manipulated CT image used as anatomical image (**b**), and SPECT image (**c**) for kernel side information

A NEMA phantom with spherical inserts (hot) and a cylindrical lung equivalent (cold insert) was filled with $^{90}$Y. Yttrium chloride in an aqueous solution of 0.1 mol/dm$^{3}$ hydrochloric acid also containing inactive yttrium ($^{89}$Y) at a concentration of 100 $\upmu$g/g. The data were acquired at the National Physical Laboratory (NPL), UK, using the triple modality scanner Mediso AnyScan Trio SCP. The NEMA phantom (Fig. 1a) contained 6 spherical inserts of different volumes and the same activity concentration. The diameter of each sphere was 10 mm, 13 mm, 17 mm, 22 mm, 28 mm and 37 mm, and the activity was 2.152± 0.003 MBq, 3.24± 0.02 MBq, 10.15± 0.06, 21.7± 0.1 MBq, 44± 0.3 MBq, 108± 1 MBq, and the cold background was filled with water. The total filled activity is then 187± 4 MBq. The SPECT data were acquired for 40 min, with 120 20 s projections. The energy window was set between 50 and 150 keV. A parallel-hole medium energy general purpose (MEGP) collimator was used. The CT image was acquired for attenuation estimation, but was also used as an anatomical prior in the HKEM and MHKEM reconstructions. The PET data were acquired for 1 h.

### Clinical data

**Fig. 2:**
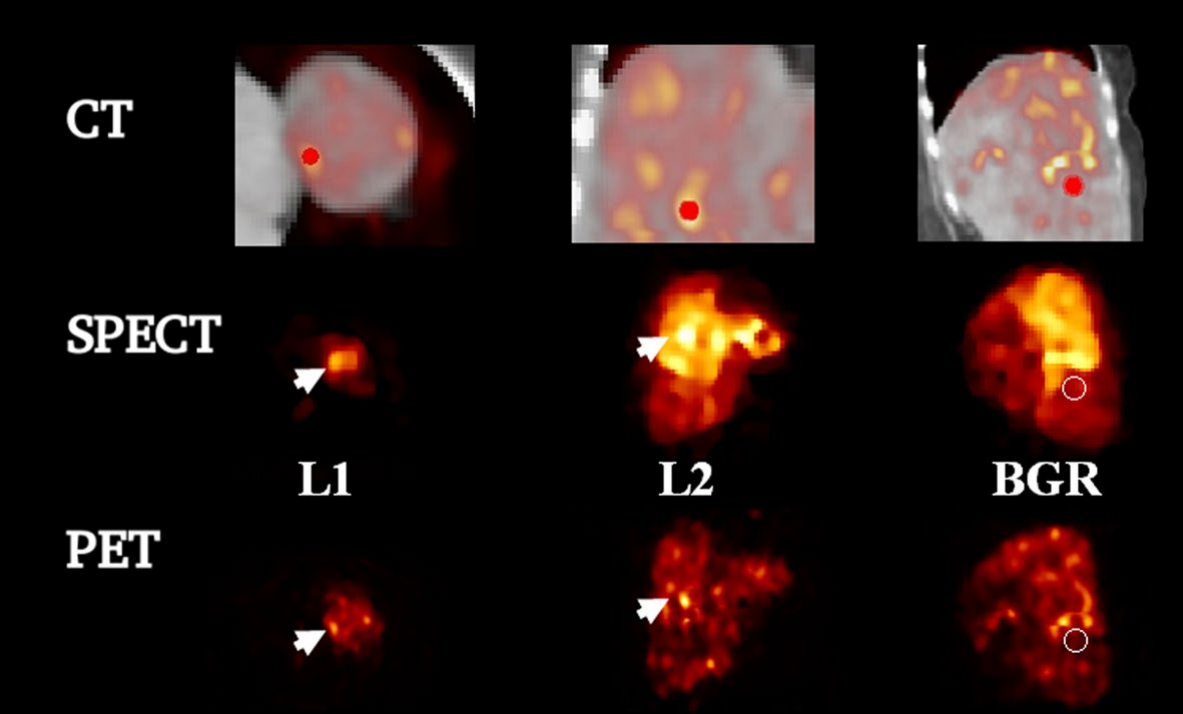
Magnified CT, SPECT and PET images for patient 1 with volume of interests. The CT and SPECT images are the one used as side information for HKEM and MHKEM. *L*1 is a lesion in the proximity of the liver surface, *L*2 is a lesion with the maximum voxel value, and BGR is the background VOI. On the top row, the segmented VOI is superimposed to the fused PET-CT image. The fused images are magnified to better show the VOIs

Data from 10 patients treated with $^{90}$Y SIRT were acquired at Oxford University Hospitals NHS Foundation Trust (OUH), UK, using the GE Discovery 670 for SPECT/CT data and GE Discovery 710 for PET/CT data. The cases were a mixture of treatments with SIR-Spheres (SIRTeX) ($n = 6$) and TheraSpheres (BTG) ($n = 4$) [29]. Images were acquired approximately 18 h following SIRT administration with PET/CT followed by SPECT/CT. The SPECT data were acquired for 30 min using a medium energy collimator and a energy window range 50–150 keV [30]. The PET data were acquired for 15 min per bed position with two bed positions acquired [29, 31]. Figure 2 shows the CT, SPECT and PET images of one patient.

### Reconstruction setup

**Fig. 3:**
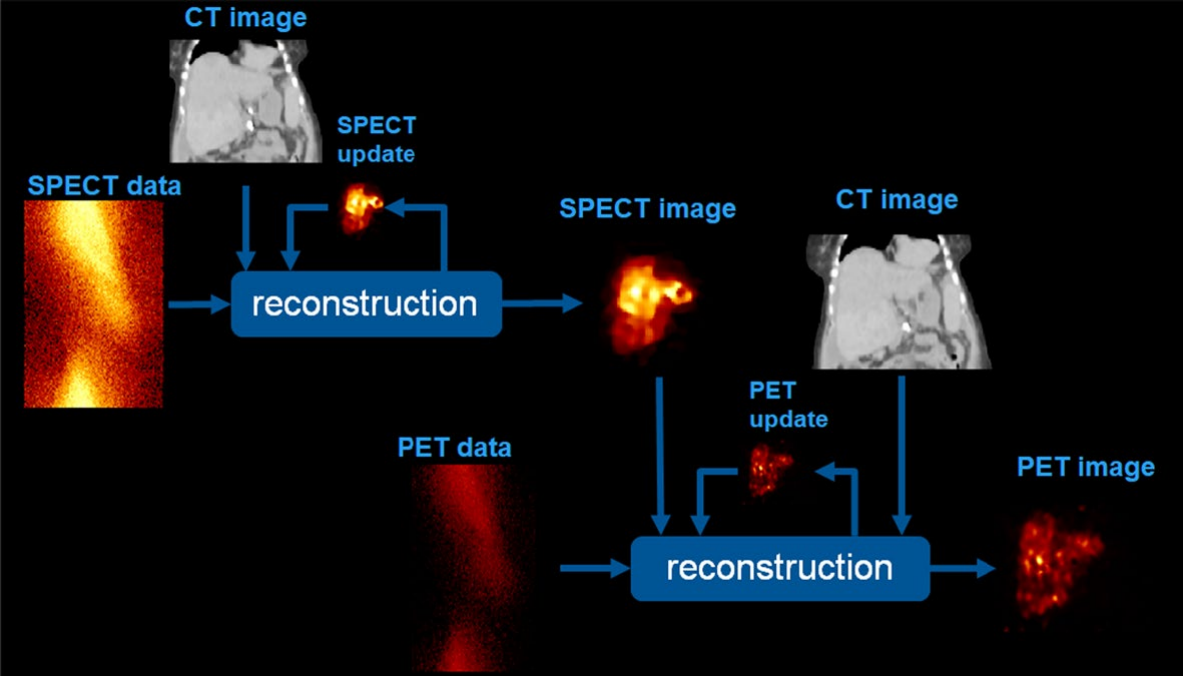
Schematic description of the triple modality reconstruction strategy, showing PET data reconstructed using PET, SPECT, CT information, where the SPECT information was obtained using SPECT/CT information

Our strategy for the triple modality reconstruction is represented schematically in Fig. 3. The bremsstrahlung data (SPECT) were first reconstructed with HKEM using the SPECT image-update and CT information as in [7]. The PET data are reconstructed using MHKEM which uses SPECT and CT images as well as the PET image estimate. Different combinations of prior images were investigated:

- SPECT, CT and PET (MHKEM)
- SPECT and PET (HKEMspect)
- CT and PET (HKEMct)
- SPECT, CT (MKEM)
- SPECT (KEMspect)
- CT (KEMct)

The HKEMspect algorithm can be considered a triple modality reconstruction method. This is because the SPECT image used in the kernel matrix was obtained using CT information.

#### Phantom data

Support for the Mediso AnyScan SCP has previously been implemented in the open source Software for Tomographic Image Reconstruction (STIR) [32–35]. The data were reconstructed using OSEM with Gaussian post-filter (with 7 mm full width half maximum (FWHM)) and no PSF resolution recovery (OSEM-noPSF), OSEM with PSF resolution recovery and Gaussian post-filter (OSEM) and with the HKEM reconstruction combinations listed above. The FWHM of the Gaussian post-filter used for OSEM was selected to give similar noise levels compared to the other method.

The images used as side information were manipulated to introduce spatial inconsistencies between PET, CT and SPECT by removing spheres, and they are reported in Fig. 1b, c. Attenuation correction factors for SPECT and PET data were estimated with STIR following the procedure in [36, 37].

Due to the low resolution of the $^{90}$Y bremsstrahlung SPECT images, the SPECT data were reconstructed using HKEM with CT side information. The SPECT image size was 128$\times$128$\times$128, while the voxel size was 4$\times$4$\times$4 mm$^3$. SPECT images were up-sampled using overlap interpolation to match the PET images with size 161$\times$161$\times$75 and voxel size 3.9$\times$3.9$\times$1.95 mm$^3$. An extensive optimization of all the kernel parameters was performed in terms of VOI recovery coefficient and coefficient of variation (CoV) in the background, and to avoid the appearance of artifacts. As a result, the number of subsets was set to 9, and the optimal kernel parameters for the SPECT and PET reconstruction are reported in Tables 1 and 2.

**Table 1:** HKEM optimal parameter values for the SPECT data

Neighbors	5 $\times$ 5 $\times$ 5
Functional iterative edge $\sigma _s$	0.1
Anatomical edge ct $\sigma _{c}$	1
Spatial distance $\sigma _{\textrm{d}s}$	5

**Table 2:** MHKEM/HKEM optimal parameter values for the PET data

Neighbors	5 $\times$ 5 $\times$ 5
Functional edge iterative PET $\sigma _p$	1
Functional edge SPECT $\sigma _{s}$	3
Anatomical edge CT $\sigma _{c}$	0.5
Spatial distance $\sigma _{dp}$	5

The analysis was carried out using segmented regions from the original CT image, as indicated in Fig. 1a, to calculate the mean recovery coefficient and the coefficient of variation (CoV) from the background VOI as follows:

1 $$\begin{aligned} \textrm{RC} = \frac{\textrm{AC}_m}{\textrm{AC}_t} \end{aligned}$$

where RC is the recovery coefficient, $\textrm{AC}_t$ is the injected activity concentration and $\textrm{AC}_m$ is the mean VOI measured activity concentration.

2 $$\begin{aligned} \textrm{CoV}= \frac{\textrm{SD}_{\textrm{bgr}}}{\textrm{Mean}_{\textrm{bgr}}}*100 \end{aligned}$$

The selected VOI is spherical with the same diameter of the insert.

#### Clinical data

The SPECT data were reconstructed using HKEM with CT information (acquired for the SPECT acquisition). The PET data were reconstructed using OSEM with Gaussian post-filter and no PSF resolution recovery, HKEM with only CT (HKEMct), HKEM with only SPECT (HKEMspect) and multiplexing HKEM (MHKEM), using both SPECT and CT images as side information (MHKEMspect-ct). The KEM options were not used in this case as the results from the NEMA phantom showed poor performance. The images used as side information are: the CT image (acquired with PET) and the SPECT image (reconstructed using HKEM with SPECT and CT as in [7]). These CT (acquired together with PET) and SPECT images are reported for one patient in Fig. 2.

The image size for SPECT was 128 $\times$ 128 $\times$ 128, while the voxel size was 4.41 $\times$ 4.41 $\times$ 4.41 mm$^3$. For PET images, the size was 256 $\times$ 256 $\times$ 84, while the voxel size was 2.13 $\times$ 2.13 $\times$ 3.27 mm$^3$. SPECT images were resampled to match the PET images.

The PET data were acquired in two bed positions, and an OSEM reconstruction was performed for each position. The combined PET OSEM image was then used to register the CT and SPECT, using niftyreg [38], which were then cropped to adapt to each bed position. Correction estimation sinograms were extracted using GE’s Duetto toolbox, while the reconstruction is performed using STIR without ToF. The PET images reconstructed using the GE toolbox with the Bayesian penalized likelihood (BPL), QClear™, a beta value of 4000, PSF resolution recovery applied, and ToF, were used as reference in the comparison. To make sure the images obtained with STIR use the same units as the one created with the vendor software, a cubic VOI including all the active voxels was used to estimate a scaling factor for the STIR images.

The analysis was carried out using spherical VOIs within tumors (Fig. 2). In particular, two different lesions were selected: a lesion that is in the proximity of the liver surface, to study the effect of the CT information on the reconstructed images, referred to as *L*1; The second lesion, *L*2, is the one with the hottest voxel, which in most cases was also the biggest lesion. Finally, a background VOI (BGR) was selected in the part of the liver with no lesions and with uniform uptake to estimate CoV. It can be challenging to select a region that is truly uniform for SIRT micro-sphere distribution in the liver; however, we used threshold-based segmentation to minimize the variation within the VOI. In addition, by comparing algorithms at similar CoV, we can make sure that every method has the same noise level. The VOIs for *L*1 and *L*2 have, respectively, a diameter of 9 and 13 mm. However, the lesions have different shapes and size, but *L*1 is always bigger than 9 mm, and *L*2 is always bigger than 13 mm. Only lesions visible in the SPECT image, the HKEM images and the QClear image were considered. The size of the VOI was chosen to fit within HKEM and QClear PET lesions.

To assess the statistical significance of the difference between algorithms, a paired *t*-test was used for each VOI. Since the *t*-test assumes normal distributions of the pair of data, a Shapiro test was also performed to test normality. All analysis was performed using R packages [39].

## Results

**Fig. 4:**
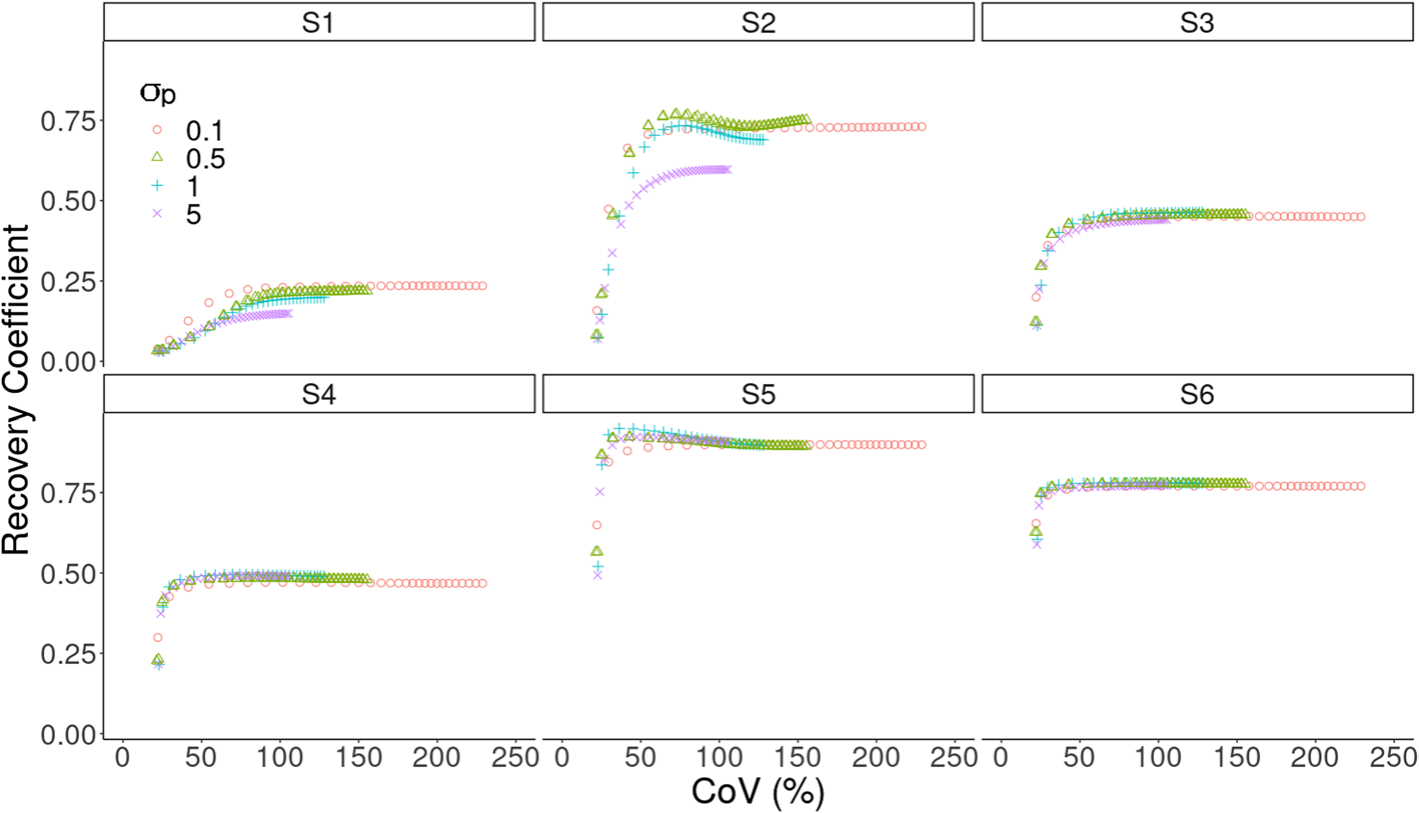
Comparison of PET images reconstructed with varying kernel parameter $\sigma _p$. S1–S6 are the VOI for the spheres numbered from the smallest to the biggest. The plot shows increasing CoV and iteration number (1–30)

**Fig. 5:**
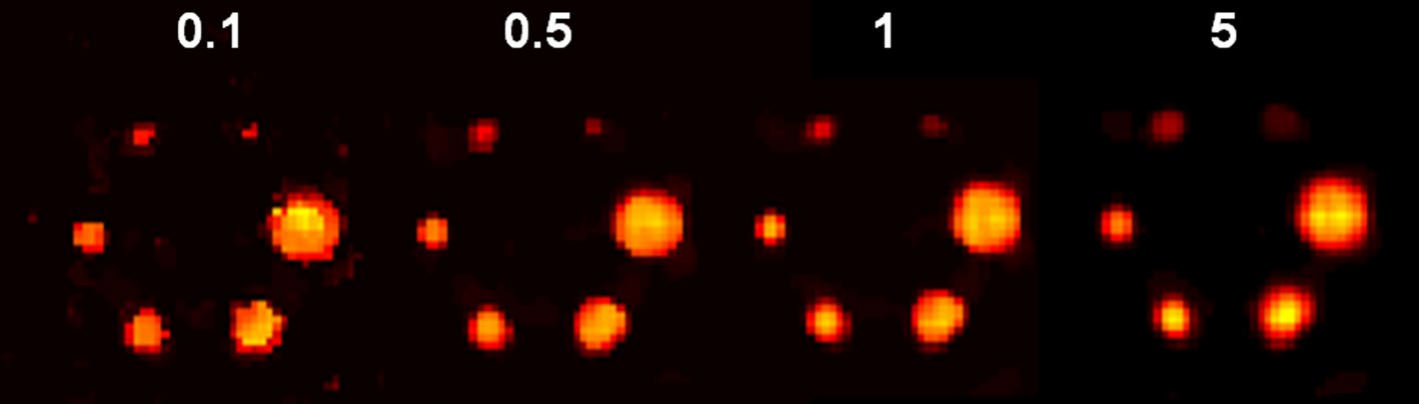
PET Images reconstructed with varying kernel parameter $\sigma _p$. Images are shown at the 10th iteration and at the same color scale

**Fig. 6:**
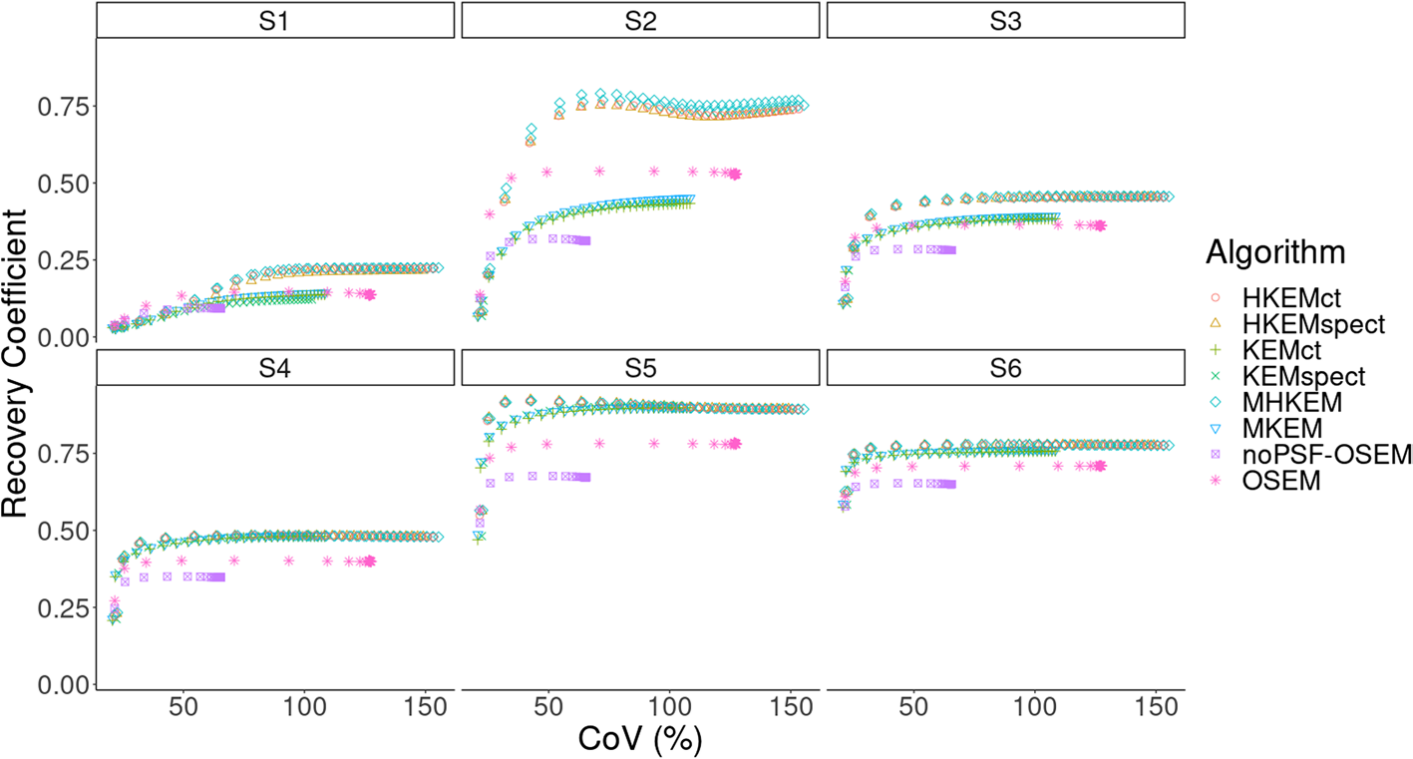
Comparison of mean recovery coefficient for each VOI between PET reconstructed images of the NEMA phantom with OSEM-noPSF, OSEM, KEMspect, KEMct, MKEM, HKEMct, HKEMspect and MHKEM. S1–S6 are the VOI for the spheres numbered from the smallest to the biggest. The plot shows increasing CoV and iteration number (1–30)

**Fig. 7:**
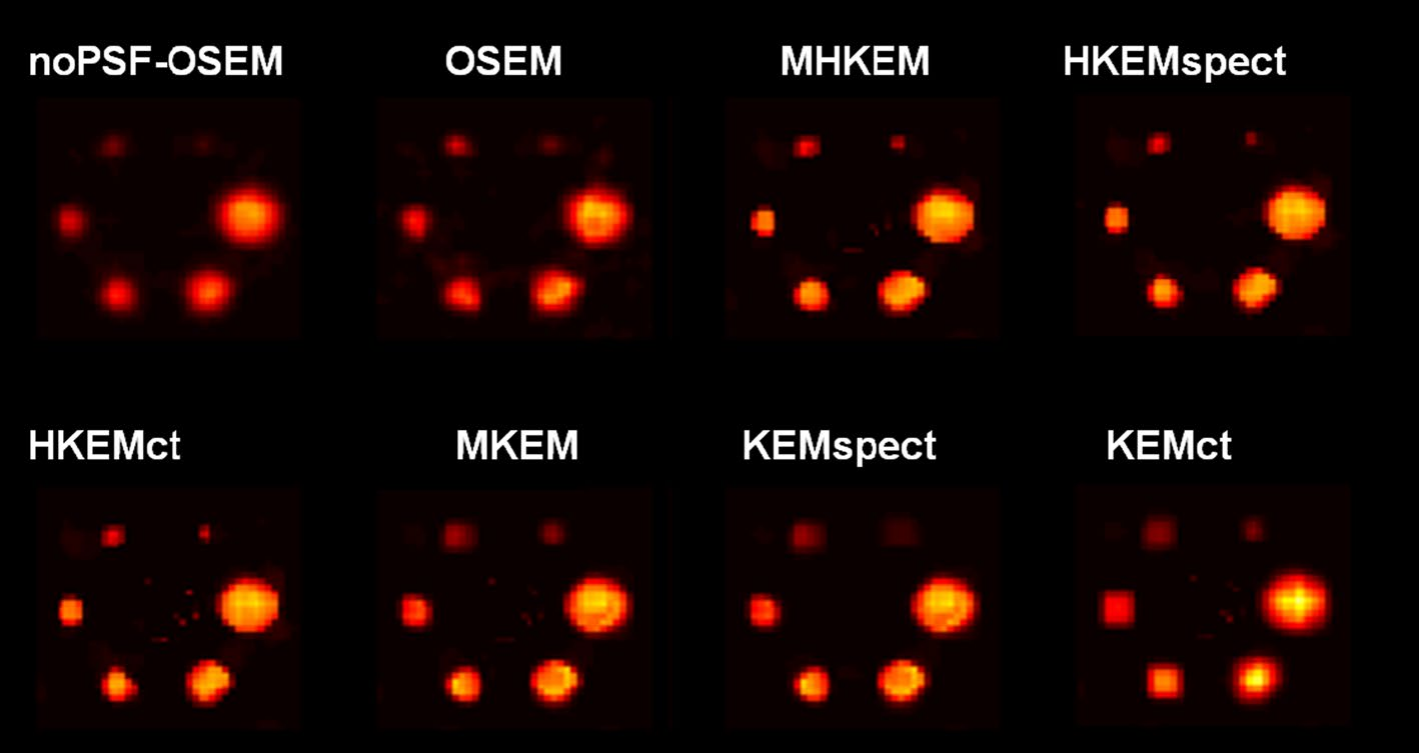
PET Reconstructed images of the NEMA phantom with OSEM-noPSF, OSEM, KEMspect, KEMct, MKEM, HKEMct, HKEMspect and MHKEM

Figure 4 reports the mean VOI recovery coefficient in each sphere of the NEMA phantom, numbered from smallest to largest. The behavior of the MHKEM algorithm at varying $\sigma _p$, which controls the strength of the PET edge preservation is also compared. All the other kernel parameters were fixed. The same procedure was then used for each kernel parameter and each algorithm. Figure 5 shows a qualitative comparison of an axial cross section demonstrating the impact of $\sigma _p$ on image quality. The value of $\sigma _p$=1 resulted to provide the best trade-off between VOI recovery coefficient and background CoV. Values of $\sigma _p$= 0.1, 0.5 and 1 provide similar mean VOI values. Nevertheless, the reconstructed images show artifacts for $\sigma _p$= 0.1 and 0.5. The algorithms with the optimized parameters are then compared in Fig. 6 in terms of VOI mean and CoV on the background. All the MHKEM/HKEM algorithms combinations are compared with OSEM and noPSF-OSEM. The reconstructed images are compared in Fig. 7.

**Fig. 8:**
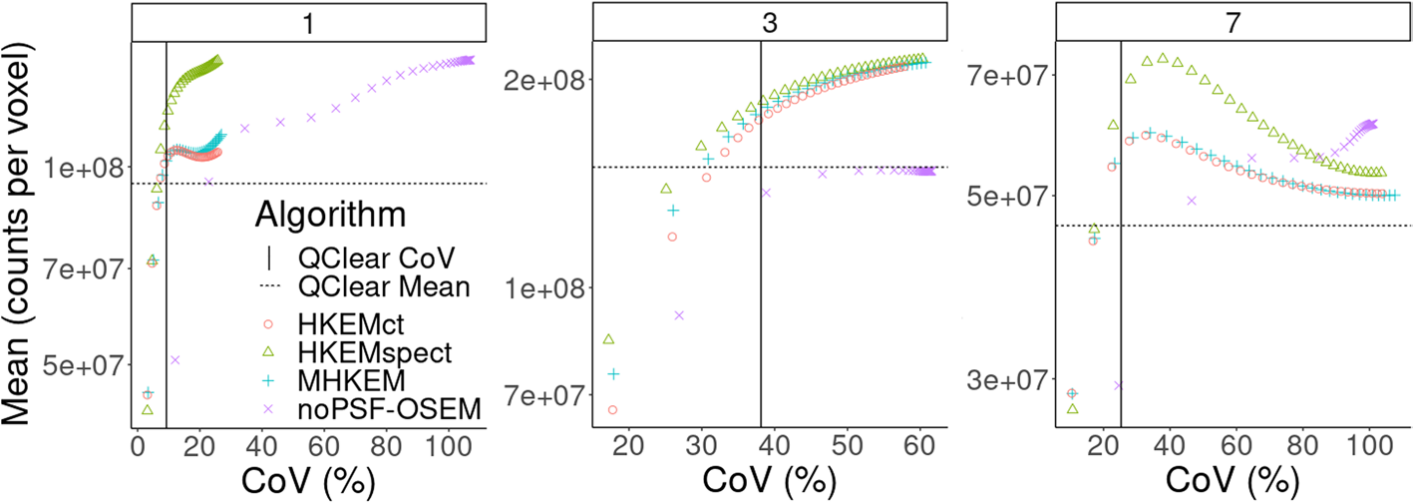
Mean lesion (*L*1) value plotted against the CoV of the background for Patient 1, 3 and 7. Each color and shape represents a different reconstruction algorithm the vertical line indicates the background CoV obtained with QClear and the dashed horizontal lines report the VOI mean value with QClear. The plot shows increasing CoV and iteration number (1–30)

**Fig. 9:**
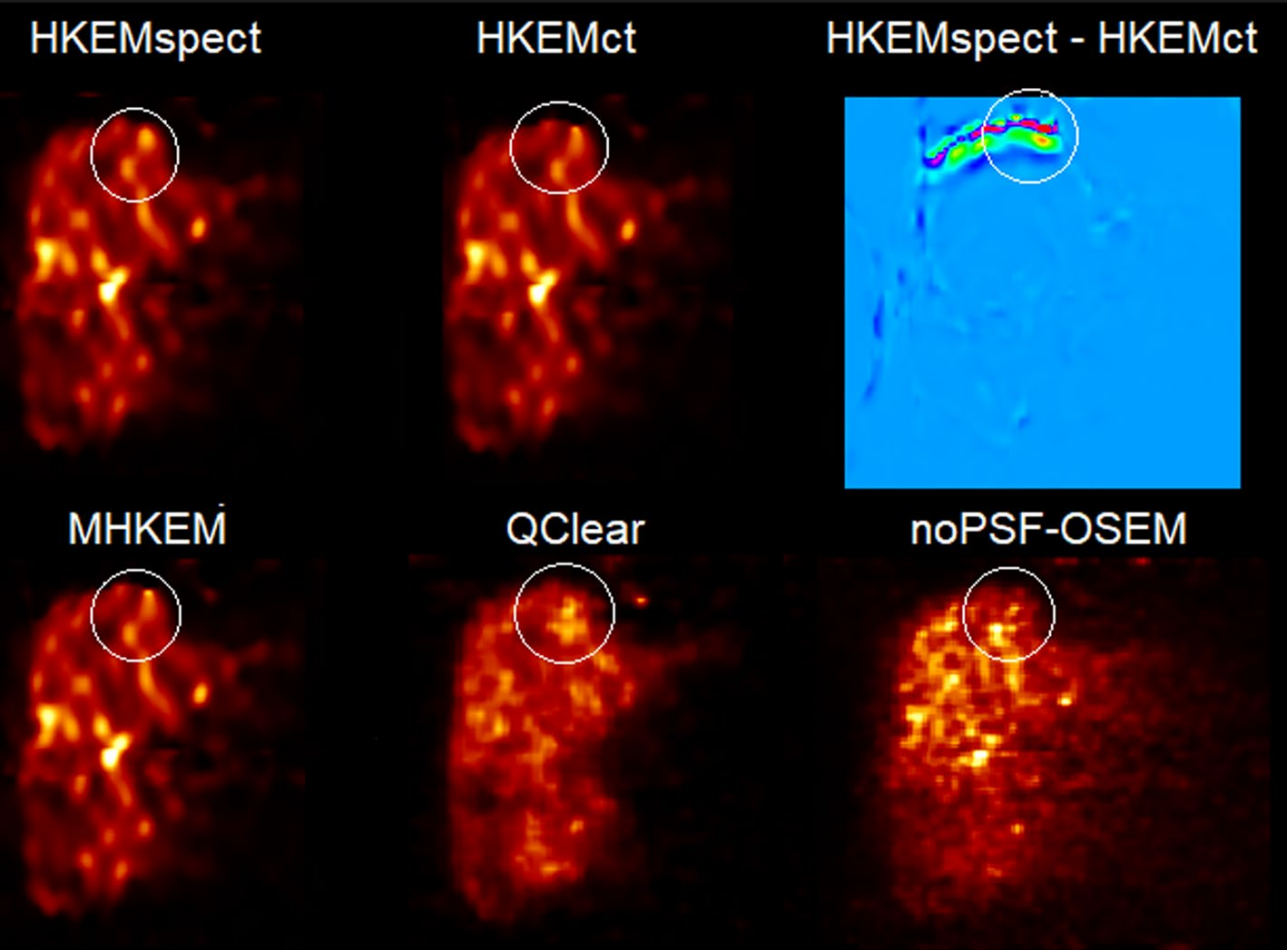
Comparison of reconstructed PET images for patient 1 reconstructed with HKEMspect, HKEMct, MHKEM, noPSF-OSEM, QClear and the difference between the images reconstructed with HKEMspect and HKEMct (red=positive, dark-blue= negative). The images are shown in the coronal view, and all use the same color scale

**Fig. 10:**
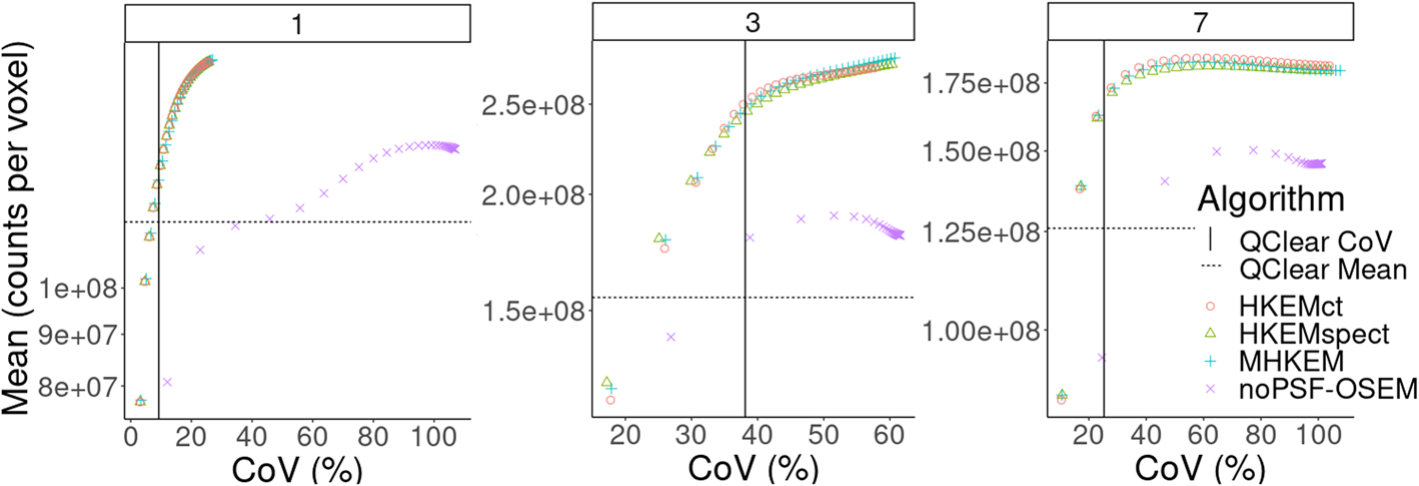
Mean lesion (*L*2) value plotted against the CoV of the background for Patient 1, 3 and 7. Each color and shape represents a different reconstruction algorithm the vertical line indicates the background CoV obtained with QClear and the dashed horizontal lines report the VOI mean value with QClear. The plot shows increasing CoV and iteration number (1–30)

**Fig. 11:**
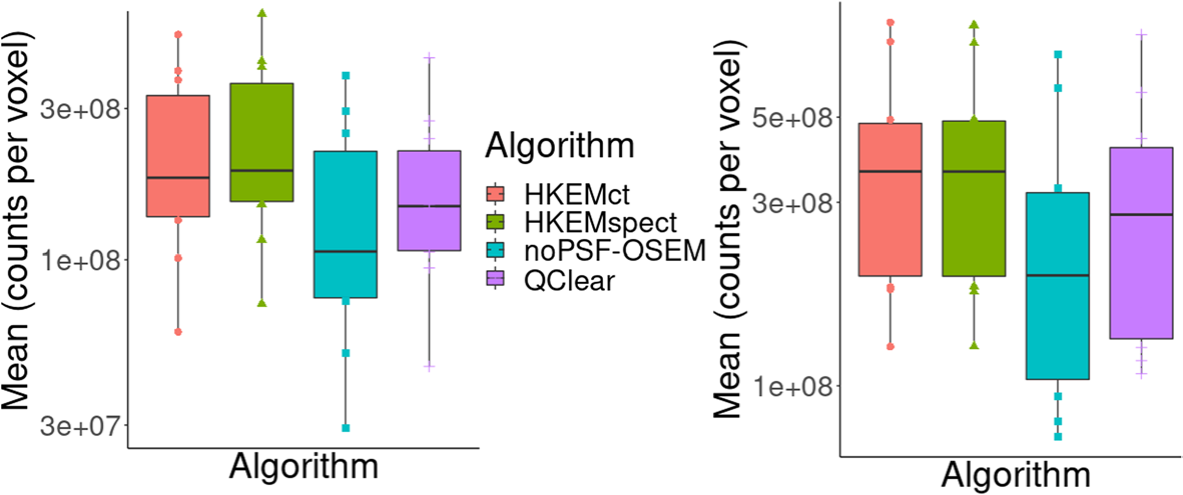
Mean lesion value distributions for all patients. Results are reported for *L*1 on left the and for *L*2 on the right. Each color and shape represents a different reconstruction algorithm

For the clinical data, the *L*1 mean VOI value plotted as a function of CoV with the progressing iterations, for three different patients, is reported in Fig. 8. The same is reported for *L*2 in Fig. 10. Each color and shape represents a different algorithm, as reported in the legend. The crossing point between the dashed horizontal line and vertical line represents QClear performance, which is the reference for the comparison. Figure 9 shows an example of image quality for all the algorithms, for one patient, and the difference between HKEMspect and HKEMct. The latter is to highlight the effect of the CT information in the proximity of the liver edges. Figure 11 shows the Mean VOI distributions among all patients for each algorithm. *L*1 is shown on the left and *L*2 on the right.

The results of a paired *t*-test are summarized in Table 3. The MHKEM algorithm was not included in the analysis because it is essentially equal to HKEMct. The results of each algorithm have been compared in pairs to investigate whether the difference obtained in this work is significant at the confidence interval of 95%. The table shows the *P*-values for *L*1 and *L*2. The *t*-test was run with Bonferroni correction.

**Fig. 12:**
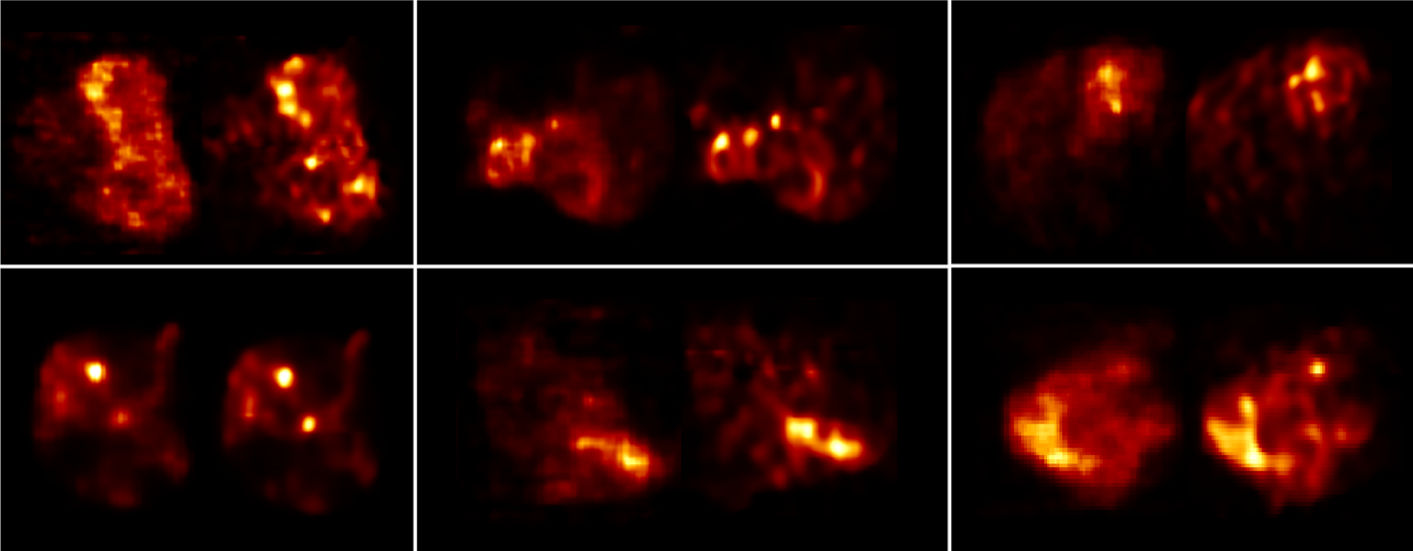
Reconstructed images with QClear (on the left of each box) and HKEMspect (on the right of each box) showing 6 different patients

Finally, to give an idea of the difference in image quality between the gold standard (with ToF) used in the clinical practice (QClear) and the HKEMspect algorithm, which provides the highest VOI values. Figure 12 shows the comparison between the image reconstructed with QClear on the left and HKEMspect on the right for 6 patients.

**Table 3:** Paired *t*-test: null hypothesis means are equal, 95% CI

Pair	*P*-value (*L*1)	*P*-value (*L*2)
HKEMspect - QClear	0.011	0.011
HKEMspect - HKEMct	0.048	1.
HKEMct - QClear	0.019	0.008
HKEMspect - noPSF-OSEM	0.012	0.005
QClear - noPSF-OSEM	0.2	0.11

## Discussion

The objective of this work was to investigate the use of various kernel-based methods for triple modality reconstruction. All these algorithms as described in [16] have hyper-parameters to tune, and the optimization of these parameters for every algorithm needed to be performed for a fair comparison. Figures 4 and 5 provide an example, for $\sigma _p$, of how the optimization needs to include quantification and image quality comparison. In fact, it may seem that quantitatively $\sigma _p = 0.1$ would give the best performance, but the noise propagates quickly with iterations, and artifacts were visible.

Although $\sigma _p = 1$ produces a slightly lower value for *S*1, it gives a smoother image and with reduced artifacts. Figure 6 shows that in general all the algorithms with side information outperform OSEM with and without PSF resolution recovery. All the algorithms incorporating the image-uptake information are providing the highest uptake for each spheres.

Previous studies, performed with HKEM using simulated data where lesions of different size are in hot or cold background [16, 22, 23, 25, 27], have shown that the HKEM algorithm provides mean VOI values that are close to the true and slightly lower than the true value. Given that all HKEM/MHKEM versions in the phantom study have shown recovery coefficient smaller than one, it could be fair to assume that the VOI values for the clinical data are also below the real value. Therefore, higher VOI values could likely mean more accurate values.

Similarly, from Fig. 7 the same results can be observed from the image quality perspective. Recall that the (manipulated) CT image used in the kernels does not have information about S2, S3 and S4. The OSEM and KEM images show low recovery in the small spheres.

For KEM, this is because the CT image used in the kernel does not have information about S2, S3 and S4. MHKEM, HKEMspect and HKEMct show similar quantitative properties. However, an artifact appears when CT information is used directly (HKEMct and MHKEM). This artifact is due to the edges of the cylindrical insert in the phantom. In light of these results, the comparison with the clinical data was not performed with the worst performing algorithms, i.e., MKEM, KEMspect and KEMct. These preliminary results show that MHKEM, which is more difficult to optimize and needs longer reconstruction time, does not provide a real benefit over HKEMspect or HKEMct. HKEMspect appears to be more robust in avoiding artifacts from the CT side information. The phantom data represents a simplistic case, and one could worry that because of the sharp boundaries of the phantom the triple modality algorithm could artificially over-sharpen tumor edges. Nevertheless, the PET and SPECT images used by HKEMspect to recover lesion activity do not have sharp edges. This by default prevents artificial over-sharpening of lesions.

Figure 8 shows the comparison between algorithms for *L*1 and three different patients. HKEMspect is consistently providing higher uptake than the other algorithms. The difference between HKEMspect and HKEMct is due to the fact that the CT information is less enforced in HKEMspect. In fact, the CT image was used to improve the SPECT image quality and not directly to improve the PET reconstruction. Therefore, HKEMspect is less affected by artifacts from CT, even though is still using triple-modality information. In MHKEM and HKEMct, the CT information has a stronger impact because the kernel matrix is directly estimated from the CT image.

When comparing images at CoV values that are closest to QClear CoV, all the algorithms using side information provide higher lesion uptake than QClear and OSEM. To support these findings, Fig. 9 shows the images for patient 1. Within the circle is *L*1. It is visible in HKEMct and MHKEM that *L*1 has been deformed, and the activity has been pushed toward the edge of the liver. The difference between HKEMspect and HKEMct highlights how the two images are very similar within the liver but different at the proximity of the liver edges.

Figure 10 shows the same analysis as Fig. 8 for the region *L*2. The outcome of this comparison still shows consistent increase for all the HKEM algorithms against OSEM and QClear, but the difference among the HKEM algorithms for *L*2 is small. This is because *L*2 is a big lesion for all patients and it is less affected by PVE. By this point, it is clear that MHKEM is not providing real quantitative benefits and is the algorithm that requires the most optimization and reconstruction time. For this reason, MHKEM is not reported in Fig. 11, where the VOI mean value distribution for all patients is showed in a box plot for *L*1 and *L*2. This plot is a confirmation of the results described above but shows the comparison for all the patients. The difference between HKEMspect and the QClear VOI values is in the range of 18–68% for *L*1 and 6–69% for *L*2. The significance of these differences was studied with a paired *t*-test. The results reported in Table 3 mean that all algorithms are significantly different between each other, except for the pair noPSF-OSEM-QClear, when looking at *L*1 values. This is also true for *L*2 except for the pair HKEMspect-HKEMct. The image quality comparison between the QClear images, used as gold standard at OUH, and the algorithm providing the highest uptake (HKEMspect), shows that triple modality reconstruction can provide smoother images, higher contrast and better definition of lesions even when ToF is not used.

The above results demonstrate that triple modality reconstruction for PET is beneficial in the context of $^{90}$Y SIRT from both a quantitative and qualitative point of view. Our results suggest that HKEMspect provides significantly higher VOI values than all other methods. Since small lesions are more visible with the HKEMspect algorithm, it could make it easier to determine whether the treatment is performing as planned. The images obtained with our method could potentially be used to plan the dose on a consecutive treatment or to investigate whether a further treatment is needed. Furthermore, accurate image-based dosimetry will help establish a dose–response relationship for better understanding the treatment outcomes when using $\beta ^-$ emitting radionuclides.

This study could be extended to other theranostic applications where PET and SPECT images are used. This investigation focused on the improvement of PET images when using SPECT information, but it is expected that SPECT images would be improved by PET information as demonstrated in [27]. For this reason, it will be important to also investigate a way to jointly reconstruct PET and SPECT data.

The results from the clinical data were obtained with two separate SPECT and PET scanners. As a consequence, a lot of attention was needed to be paid in the registration process, which could be facilitated by the use of triple modality scanners.

Other factors could play a role in degrading lesion quantification, an example is represented by respiratory motion, and lesions that are close to the lungs would be the most affected. The presence of the PET image-update in HKEM can reduce the effect of any misalignment [21]; however, motion correction techniques would likely improve our results. Having the possibility to acquire simultaneous PET-SPECT data could make the task easier. It is worth noting that although our results are promising and consistent, they are obtained using a relatively small sample of patients and more clinical data need to be investigated.

## Conclusion

This study proposes the use of triple modality information PET, SPECT, CT for the improvement of both image quality and quantification of PET images in the application of SIRT using Y90 micro-spheres. The phantom and the clinical data results are in agreement and show significant increase in VOI value when using a triple modality reconstruction compared to the algorithms used in the hospital. Images with the HKEMspect algorithm show better definition of lesions. Given the improvement in accuracy provided with the phantom, it is fair to assume that our triple modality reconstruction could help enabling personalized treatment planning and provide more accurate monitoring of the treatment.

## Data Availability

The triple modality acquisition of the NEMA data is available at https://osf.io/pcfb4/.

## Abbreviations

CT: Computed tomography
OUH: Oxford University Hospitals NHS Foundation Trust
SPECT: Photon emission computed tomography
PET: Positron emission tomography
$$^{90}$$Y: Yttrium-90
PVE: Partial volume effect
PSMA: Prostate-specific membrane antigen
MAA: Macro-aggregated albumin
HCC: Hepatocellular carcinoma
SIRT: Selective internal radiation therapy
HKEM: Hybrid kernelized expectation maximization
MHKEM: Multiplexing hybrid kernelized expectation maximization
VOI: Volume of interest
CoV: Coefficient of variation
STIR: Software for Tomographic Image Reconstruction
MEGP: Medium energy general purpose
PSF: Point spread function
OSEM: Ordered subsets expectation maximization
bgr: Background

## Code availability

All reconstruction algorithms used in the manuscript are freely available in https://github.com/UCL/STIR.git and the synergistic image reconstruction framework (https://github.com/SyneRBI/SIRF.git).
